# 3,4-Diaminopyridinium 2-carboxy-4,6-dinitrophenolate

**DOI:** 10.1107/S1600536810038936

**Published:** 2010-10-09

**Authors:** Madhukar Hemamalini, Hoong-Kun Fun

**Affiliations:** a X-ray Crystallography Unit, School of Physics, Universiti Sains Malaysia, 11800 USM, Penang, Malaysia

## Abstract

In the title salt, C_5_H_8_N_3_^+^·C_7_H_3_N_2_O_7_^−^, the pyridine N atom of the 3,4-diamino­pyridine mol­ecule is protonated. The 3,5-dinitro­salicylate anion shows whole-mol­ecule disorder over two orientations with a refined occupancy ratio of 0.875 (4): 0.125 (4). In the crystal, the cations and anions are connected by inter­molecular N—H⋯O and C—H⋯O hydrogen bonds, forming a three-dimensional network.

## Related literature

For applications of diamino­pyridine, see: Abu Zuhri & Cox (1989 ▶); Inuzuka & Fujimoto (1990 ▶); El-Mossalamy (2001 ▶). For related structures, see: Rubin-Preminger & Englert (2007 ▶); Koleva *et al.* (2007 ▶); Koleva *et al.* (2008 ▶). For reference bond-length data, see: Allen *et al.* (1987 ▶). For the stability of the temperature controller used in the data collection, see: Cosier & Glazer (1986 ▶).
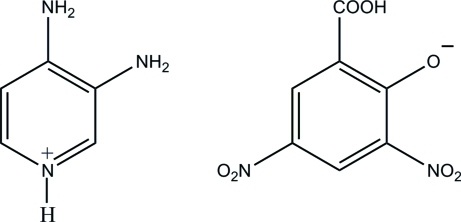


## Experimental

### 

#### Crystal data

C_5_H_8_N_3_^+^·C_7_H_3_N_2_O_7_^−^
*M* *_r_* = 337.26
Monoclinic, 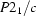

*a* = 9.1187 (4) Å
*b* = 11.3569 (5) Å
*c* = 13.1343 (6) Å
β = 98.204 (4)°
*V* = 1346.27 (10) Å^3^
*Z* = 4
Mo *K*α radiation
μ = 0.14 mm^−1^
*T* = 100 K
0.52 × 0.11 × 0.10 mm

#### Data collection

Bruker SMART APEXII CCD diffractometer
Absorption correction: multi-scan (*SADABS*; Bruker, 2009 ▶) *T*_min_ = 0.931, *T*_max_ = 0.986
10195 measured reflections
2785 independent reflections
1979 reflections with *I* > 2σ(*I*)
*R*_int_ = 0.064

#### Refinement

*R*[*F* ^2^ > 2σ(*F* ^2^)] = 0.070
*wR*(*F* ^2^) = 0.162
*S* = 1.12
2785 reflections
287 parameters
526 restraints
H atoms treated by a mixture of independent and constrained refinement
Δρ_max_ = 0.46 e Å^−3^
Δρ_min_ = −0.28 e Å^−3^

### 

Data collection: *APEX2* (Bruker, 2009 ▶); cell refinement: *SAINT* (Bruker, 2009 ▶); data reduction: *SAINT*; program(s) used to solve structure: *SHELXTL* (Sheldrick, 2008 ▶); program(s) used to refine structure: *SHELXTL*; molecular graphics: *SHELXTL*; software used to prepare material for publication: *SHELXTL* and *PLATON* (Spek, 2009 ▶).

## Supplementary Material

Crystal structure: contains datablocks global, I. DOI: 10.1107/S1600536810038936/hb5660sup1.cif

Structure factors: contains datablocks I. DOI: 10.1107/S1600536810038936/hb5660Isup2.hkl

Additional supplementary materials:  crystallographic information; 3D view; checkCIF report

## Figures and Tables

**Table 1:** Hydrogen-bond geometry (Å, °)

*D*—H⋯*A*	*D*—H	H⋯*A*	*D*⋯*A*	*D*—H⋯*A*
N1—H1N1⋯O1	1.07	1.76	2.753 (4)	153
N2—H2N2⋯O6^i^	0.89	2.24	3.120 (4)	171
N2—H1N2⋯O3^ii^	1.03	2.11	3.026 (5)	146
N3—H1N3⋯O6^i^	0.89	2.36	3.104 (4)	142
N3—H2N3⋯O5^iii^	1.00	2.24	3.217 (5)	163
C6—H6*A*⋯O3^iv^	0.93	2.56	3.299 (6)	136

Symmetry codes: (i) 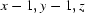; (ii) 
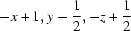
; (iii) 
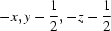
; (iv) 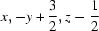.

**Fig. 1.**
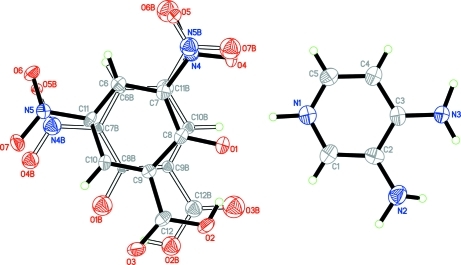
The asymmetric unit of the title compound. Displacement ellipsoids are drawn at the 50% probability level. All disorder components are shown.

**Fig. 2.**
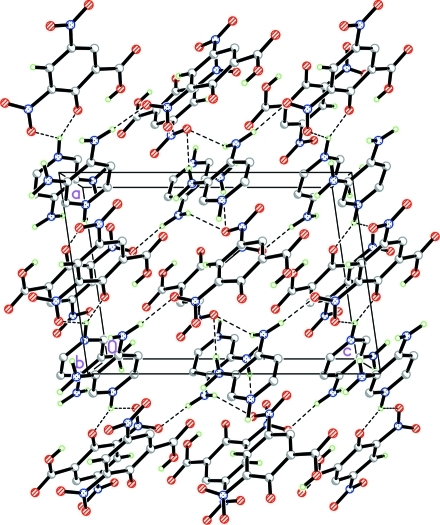
The crystal packing of the title compound, showing a hydrogen-bonded (dashed lines) network. H atoms not involved in the interactions have been omitted for clarity.

## supplementary crystallographic information

### Comment

Diaminopyridine have an important role in the preparation of aromatic azo dyes (Abu Zuhri & Cox, 1989; Inuzuka & Fujimoto, 1990) and in many polarographic investigations (El-Mossalamy, 2001). The crystal structure of 3,4-diaminopyridine (Rubin-Preminger & Englert, 2007), 3,4-diaminopyridinium hydrogen squarate (Koleva *et al.*, 2007) and 3,4-diaminopyridinium hydrogen tartarate (Koleva *et al.*, 2008) have been reported in the literature. 3,5-Dinitrosalicylic acid (DNSA) has proved to be effective as a proton-donating acid species for stabilizing crystalline salts of Lewis bases. Since our aim is to study some interesting hydrogen-bonding interactions, the synthesis and structure of the title compound (I) is presented here.

The asymmetric unit of (I) (Fig 1), contains a protonated 3,4-diaminopyridinium cation and a 3,5-dinitrosalicylate anion. The bond lengths (Allen *et al.*, 1987) and angles are normal. In the 3,4-diaminopyridinium cation (the proton transfer from the hydroxyl group of the anion), protonation of the N1 atom leads to a slight increase in the C1—N1—C5 angle to 122.1 (3)°, compared to 115.69 (19)° in 3,4-diaminopyridine (Rubin-Preminger & Englert, 2007). The whole 3,5-dinitrosalicylate anion is disordered over two positions with a refined occupancy ratio of 0.886 (4): 0.114 (4). Excluding amino group, the pyridine is planar, with a maximum deviation of 0.010 (3) Å for atom C2.

In the crystal structure (Fig. 2), there is an intramolecular O2—H2···O1 hydrogen bond in the 3,5-dinitrosalicylate anion, which generates an *S*(6) (Bernstein *et al.*, 1995) ring motif.  Furthermore, the cations and anions are connected by intermolecular strong N1—H1N1···O1; N2—H2N2···O6; N2—H1N2···O3; N3—H1N3···O6; N3—H2N3···O5 and weak C6—H6A···O3 hydrogen bonds, forming a three-dimensional network.

### Experimental

A hot methanol solution (20 ml) of 3,4-diaminopyridine (27 mg, Aldrich) and 3,5-dinitrosalicylic acid (57 mg, Merck) were mixed and warmed over a heating magnetic stirrer hotplate for a few minutes. The resulting solution was allowed to cool slowly at room temperature and crystals of the title compound appeared after a few days.

### Refinement

All the H atoms were positioned geometrically [C–H = 0.93 Å; N–H = 0.8875–1.0684 Å] and were refined using a riding model, with *U*_iso_(H) = 1.2 *U*_eq_(C,N,O).  The whole 3,5-dinitrosalicylate anion is disordered over two positions with a refined ratio of 0.886 (4): 0.114 (4). In the final difference Fourier map, the highest peak and the deepest hole are 1.24 Å and 0.62 Å from H1N2 and C5, respectively.

### Crystal data

**Table:**

C_5_H_8_N_3_^+^·C_7_H_3_N_2_O_7_^−^	*F*(000) = 696
*M**_r_* = 337.26	*D*_x_ = 1.664 Mg m^−^^3^
Monoclinic, *P*2_1_/*c*	Mo *K*α radiation, λ = 0.71073 Å
Hall symbol: -P 2ybc	Cell parameters from 2307 reflections
*a* = 9.1187 (4) Å	θ = 2.4–28.6°
*b* = 11.3569 (5) Å	µ = 0.14 mm^−^^1^
*c* = 13.1343 (6) Å	*T* = 100 K
β = 98.204 (4)°	Needle, yellow
*V* = 1346.27 (10) Å^3^	0.52 × 0.11 × 0.10 mm
*Z* = 4	

### Data collection

**Table:**

Bruker SMART APEXII CCD area-detector diffractometer	2785 independent reflections
Radiation source: fine-focus sealed tube	1979 reflections with *I* > 2σ(*I*)
graphite	*R*_int_ = 0.064
φ and ω scans	θ_max_ = 26.5°, θ_min_ = 2.3°
Absorption correction: multi-scan (*SADABS*; Bruker, 2009)	*h* = −11→11
*T*_min_ = 0.931, *T*_max_ = 0.986	*k* = −14→14
10195 measured reflections	*l* = −16→16

### Refinement

**Table:**

Refinement on *F*^2^	Primary atom site location: structure-invariant direct methods
Least-squares matrix: full	Secondary atom site location: difference Fourier map
*R*[*F*^2^ > 2σ(*F*^2^)] = 0.070	Hydrogen site location: inferred from neighbouring sites
*wR*(*F*^2^) = 0.162	H-atom parameters constrained
*S* = 1.12	*w* = 1/[σ^2^(*F*_o_^2^) + (0.0439*P*)^2^ + 2.4565*P*] where *P* = (*F*_o_^2^ + 2*F*_c_^2^)/3
2785 reflections	(Δ/σ)_max_ < 0.001
279 parameters	Δρ_max_ = 0.46 e Å^−^^3^
526 restraints	Δρ_min_ = −0.28 e Å^−^^3^

### Special details

**Table:**

Experimental. The crystal was placed in the cold stream of an Oxford Cryosystems Cobra open-flow nitrogen cryostat (Cosier & Glazer, 1986) operating at 100.0 (1) K.
Geometry. All s.u.'s (except the s.u. in the dihedral angle between two l.s. planes) are estimated using the full covariance matrix. The cell s.u.'s are taken into account individually in the estimation of s.u.'s in distances, angles and torsion angles; correlations between s.u.'s in cell parameters are only used when they are defined by crystal symmetry. An approximate (isotropic) treatment of cell s.u.'s is used for estimating s.u.'s involving l.s. planes.
Refinement. Refinement of F^2^ against ALL reflections. The weighted R-factor wR and goodness of fit S are based on F^2^, conventional R-factors R are based on F, with F set to zero for negative F^2^. The threshold expression of F^2^ > 2σ(F^2^) is used only for calculating R-factors(gt) etc. and is not relevant to the choice of reflections for refinement. R-factors based on F^2^ are statistically about twice as large as those based on F, and R- factors based on ALL data will be even larger.

### Fractional atomic coordinates and isotropic or equivalent isotropic displacement parameters (Å^2^)

**Table:**

	*x*	*y*	*z*	*U*_iso_*/*U*_eq_	Occ. (<1)
N1	0.1346 (3)	0.4299 (2)	−0.0435 (2)	0.0286 (7)	
H1N1	0.2282	0.4863	−0.0241	0.034*	
N2	0.0269 (3)	0.1468 (3)	0.0541 (2)	0.0370 (8)	
H2N2	−0.0550	0.1048	0.0573	0.044*	
H1N2	0.1048	0.1417	0.1192	0.044*	
N3	−0.1784 (3)	0.1759 (2)	−0.1255 (2)	0.0279 (7)	
H1N3	−0.1717	0.1109	−0.0876	0.034*	
H2N3	−0.2376	0.1940	−0.1942	0.034*	
C1	0.1297 (4)	0.3365 (3)	0.0206 (3)	0.0283 (8)	
H1A	0.1953	0.3325	0.0816	0.034*	
C2	0.0275 (3)	0.2469 (3)	−0.0045 (3)	0.0274 (8)	
C3	−0.0744 (3)	0.2571 (3)	−0.0968 (2)	0.0253 (7)	
C4	−0.0643 (4)	0.3579 (3)	−0.1599 (3)	0.0275 (8)	
H4A	−0.1299	0.3671	−0.2204	0.033*	
C5	0.0411 (4)	0.4406 (3)	−0.1315 (3)	0.0298 (8)	
H5A	0.0486	0.5054	−0.1737	0.036*	
O1	0.3734 (3)	0.5460 (2)	0.06336 (19)	0.0242 (7)	0.886 (4)
O2	0.5011 (3)	0.4686 (2)	0.2297 (2)	0.0278 (7)	0.886 (4)
H2	0.4413	0.4777	0.1774	0.033*	0.886 (4)
O3	0.6962 (5)	0.5623 (2)	0.3110 (3)	0.0274 (9)	0.886 (4)
O4	0.2470 (6)	0.6533 (5)	−0.1085 (5)	0.0309 (11)	0.886 (4)
O5	0.3768 (6)	0.7848 (5)	−0.1745 (3)	0.0242 (10)	0.886 (4)
O6	0.7238 (3)	1.0170 (3)	0.0450 (3)	0.0246 (7)	0.886 (4)
O7	0.8474 (3)	0.9354 (3)	0.1809 (2)	0.0270 (7)	0.886 (4)
N4	0.3541 (10)	0.7207 (7)	−0.1015 (5)	0.0204 (9)	0.886 (4)
N5	0.7468 (3)	0.9350 (3)	0.1074 (3)	0.0191 (7)	0.886 (4)
C6	0.5497 (8)	0.8229 (5)	0.0041 (4)	0.0177 (14)	0.886 (4)
H6A	0.5442	0.8801	−0.0470	0.021*	0.886 (4)
C7	0.4568 (12)	0.7259 (7)	−0.0067 (6)	0.0182 (12)	0.886 (4)
C8	0.4606 (6)	0.6350 (4)	0.0699 (4)	0.0179 (9)	0.886 (4)
C9	0.5741 (4)	0.6491 (3)	0.1573 (3)	0.0171 (8)	0.886 (4)
C10	0.6650 (4)	0.7456 (3)	0.1681 (3)	0.0162 (7)	0.886 (4)
H10A	0.7364	0.7530	0.2259	0.019*	0.886 (4)
C11	0.6506 (5)	0.8326 (4)	0.0926 (3)	0.0158 (8)	0.886 (4)
C12	0.5956 (4)	0.5570 (3)	0.2394 (3)	0.0210 (8)	0.886 (4)
O1B	0.703 (2)	0.7054 (19)	0.2650 (14)	0.037 (6)*	0.114 (4)
O2B	0.598 (2)	0.512 (2)	0.2943 (15)	0.034 (6)*	0.114 (4)
H2B	0.6584	0.5652	0.2956	0.01 (17)*	0.114 (4)
O3B	0.409 (3)	0.4439 (19)	0.1805 (19)	0.051 (7)*	0.114 (4)
O4B	0.833 (2)	0.892 (2)	0.2101 (17)	0.028 (6)*	0.114 (4)
O5B	0.722 (3)	0.995 (2)	0.0788 (19)	0.013 (6)*	0.114 (4)
O6B	0.375 (7)	0.811 (4)	−0.176 (4)	0.045 (16)*	0.114 (4)
O7B	0.250 (6)	0.660 (5)	−0.132 (4)	0.044 (15)*	0.114 (4)
N4B	0.733 (3)	0.908 (2)	0.138 (2)	0.032 (7)*	0.114 (4)
N5B	0.353 (10)	0.733 (7)	−0.115 (4)	0.032 (7)*	0.114 (4)
C6B	0.537 (6)	0.824 (4)	0.018 (3)	0.011 (6)*	0.114 (4)
H6BA	0.5362	0.8931	−0.0200	0.013*	0.114 (4)
C7B	0.633 (4)	0.811 (3)	0.110 (2)	0.011 (6)*	0.114 (4)
C8B	0.620 (3)	0.714 (2)	0.1781 (16)	0.007 (6)*	0.114 (4)
C9B	0.520 (3)	0.6226 (19)	0.1401 (17)	0.014 (5)*	0.114 (4)
C10B	0.432 (5)	0.633 (3)	0.049 (3)	0.014 (5)*	0.114 (4)
H10B	0.3639	0.5746	0.0275	0.016*	0.114 (4)
C11B	0.442 (12)	0.732 (7)	−0.014 (5)	0.026 (6)*	0.114 (4)
C12B	0.499 (3)	0.521 (2)	0.2106 (18)	0.026 (6)*	0.114 (4)

### Atomic displacement parameters (Å^2^)

**Table:**

	*U*^11^	*U*^22^	*U*^33^	*U*^12^	*U*^13^	*U*^23^
N1	0.0268 (15)	0.0294 (15)	0.0305 (16)	0.0010 (13)	0.0071 (13)	−0.0046 (12)
N2	0.0307 (15)	0.0329 (17)	0.0469 (19)	−0.0038 (14)	0.0036 (14)	0.0063 (14)
N3	0.0288 (14)	0.0247 (14)	0.0296 (15)	−0.0042 (12)	0.0017 (12)	0.0005 (12)
C1	0.0268 (17)	0.0239 (17)	0.037 (2)	0.0027 (15)	0.0138 (15)	−0.0011 (15)
C2	0.0254 (17)	0.0312 (18)	0.0262 (17)	0.0103 (15)	0.0055 (14)	−0.0011 (14)
C3	0.0219 (16)	0.0277 (18)	0.0282 (18)	0.0005 (14)	0.0098 (14)	−0.0089 (14)
C4	0.0260 (16)	0.0239 (17)	0.0346 (19)	0.0016 (14)	0.0110 (15)	−0.0036 (14)
C5	0.0318 (18)	0.0268 (18)	0.0319 (19)	0.0035 (15)	0.0089 (15)	0.0015 (14)
O1	0.0243 (13)	0.0203 (13)	0.0282 (14)	−0.0050 (11)	0.0047 (11)	−0.0006 (10)
O2	0.0340 (15)	0.0211 (15)	0.0283 (15)	−0.0031 (12)	0.0053 (12)	0.0083 (12)
O3	0.0300 (15)	0.0260 (19)	0.0258 (16)	0.0037 (12)	0.0021 (15)	0.0078 (11)
O4	0.0240 (18)	0.034 (2)	0.033 (3)	−0.0167 (12)	−0.0016 (18)	0.0034 (19)
O5	0.0281 (19)	0.024 (2)	0.0201 (18)	−0.0091 (18)	0.0016 (11)	0.0047 (15)
O6	0.0303 (16)	0.0173 (15)	0.0266 (18)	−0.0026 (11)	0.0051 (14)	0.0060 (13)
O7	0.0245 (14)	0.0227 (15)	0.0308 (16)	−0.0050 (12)	−0.0065 (12)	−0.0017 (13)
N4	0.0193 (16)	0.023 (3)	0.020 (2)	−0.0003 (18)	0.0050 (17)	−0.0003 (14)
N5	0.0198 (16)	0.0176 (18)	0.0203 (18)	0.0005 (14)	0.0038 (14)	−0.0027 (15)
C6	0.019 (2)	0.0193 (19)	0.016 (2)	0.0018 (15)	0.009 (2)	−0.0020 (16)
C7	0.016 (3)	0.020 (2)	0.020 (2)	0.002 (2)	0.0068 (14)	−0.0020 (14)
C8	0.018 (3)	0.0159 (17)	0.021 (3)	0.0011 (16)	0.0086 (17)	−0.0020 (16)
C9	0.018 (2)	0.0158 (18)	0.0183 (17)	0.0026 (16)	0.0059 (14)	0.0005 (14)
C10	0.0153 (17)	0.0164 (18)	0.0182 (18)	−0.0011 (16)	0.0067 (14)	−0.0041 (14)
C11	0.018 (2)	0.011 (2)	0.020 (2)	−0.0012 (14)	0.0103 (16)	−0.0006 (14)
C12	0.0255 (19)	0.0153 (16)	0.024 (2)	0.0030 (15)	0.0096 (16)	0.0010 (15)

### Geometric parameters (Å, °)

**Table:**

N1—C5	1.341 (4)	C6—C7	1.384 (5)
N1—C1	1.358 (4)	C6—H6A	0.9300
N1—H1N1	1.0684	C7—C8	1.438 (5)
N2—C2	1.373 (4)	C8—C9	1.441 (5)
N2—H2N2	0.8919	C9—C10	1.369 (5)
N2—H1N2	1.0329	C9—C12	1.494 (5)
N3—C3	1.338 (4)	C10—C11	1.393 (5)
N3—H1N3	0.8875	C10—H10A	0.9300
N3—H2N3	1.0043	O1B—C8B	1.281 (16)
C1—C2	1.387 (5)	O2B—C12B	1.322 (16)
C1—H1A	0.9300	O2B—H2B	0.8200
C2—C3	1.423 (4)	O3B—C12B	1.229 (17)
C3—C4	1.424 (5)	O4B—N4B	1.230 (17)
C4—C5	1.357 (5)	O5B—N4B	1.249 (17)
C4—H4A	0.9300	O6B—N5B	1.237 (18)
C5—H5A	0.9300	O7B—N5B	1.246 (18)
O1—C8	1.282 (5)	N4B—C7B	1.445 (16)
O2—C12	1.317 (4)	N5B—C11B	1.454 (17)
O2—H2	0.8200	C6B—C11B	1.386 (18)
O3—C12	1.219 (6)	C6B—C7B	1.388 (18)
O4—N4	1.234 (4)	C6B—H6BA	0.9300
O5—N4	1.244 (5)	C7B—C8B	1.436 (16)
O6—N5	1.239 (5)	C8B—C9B	1.424 (16)
O7—N5	1.233 (4)	C9B—C10B	1.348 (16)
N4—C7	1.449 (5)	C9B—C12B	1.507 (16)
N5—C11	1.453 (5)	C10B—C11B	1.398 (18)
C6—C11	1.381 (5)	C10B—H10B	0.9300
			
C5—N1—C1	122.1 (3)	C10—C9—C8	121.8 (3)
C5—N1—H1N1	122.6	C10—C9—C12	118.0 (3)
C1—N1—H1N1	114.6	C8—C9—C12	120.2 (3)
C2—N2—H2N2	122.6	C9—C10—C11	120.0 (3)
C2—N2—H1N2	116.9	C9—C10—H10A	120.0
H2N2—N2—H1N2	114.3	C11—C10—H10A	120.0
C3—N3—H1N3	115.2	C6—C11—C10	121.8 (4)
C3—N3—H2N3	112.3	C6—C11—N5	119.6 (4)
H1N3—N3—H2N3	131.3	C10—C11—N5	118.6 (3)
N1—C1—C2	120.4 (3)	O3—C12—O2	121.5 (3)
N1—C1—H1A	119.8	O3—C12—C9	122.0 (3)
C2—C1—H1A	119.8	O2—C12—C9	116.5 (3)
N2—C2—C1	122.0 (3)	C12B—O2B—H2B	109.5
N2—C2—C3	119.4 (3)	O4B—N4B—O5B	126 (2)
C1—C2—C3	118.5 (3)	O4B—N4B—C7B	116.9 (19)
N3—C3—C2	122.3 (3)	O5B—N4B—C7B	116.5 (18)
N3—C3—C4	119.5 (3)	O6B—N5B—O7B	123 (3)
C2—C3—C4	118.3 (3)	O6B—N5B—C11B	119 (2)
C5—C4—C3	119.9 (3)	O7B—N5B—C11B	118 (3)
C5—C4—H4A	120.1	C11B—C6B—C7B	118.0 (19)
C3—C4—H4A	120.1	C11B—C6B—H6BA	121.0
N1—C5—C4	120.8 (3)	C7B—C6B—H6BA	121.0
N1—C5—H5A	119.6	C6B—C7B—C8B	121.6 (17)
C4—C5—H5A	119.6	C6B—C7B—N4B	115.9 (18)
C12—O2—H2	109.5	C8B—C7B—N4B	121.9 (17)
O4—N4—O5	121.5 (4)	O1B—C8B—C9B	121.6 (16)
O4—N4—C7	119.8 (4)	O1B—C8B—C7B	121.6 (17)
O5—N4—C7	118.7 (4)	C9B—C8B—C7B	116.4 (15)
O7—N5—O6	123.5 (3)	C10B—C9B—C8B	120.9 (16)
O7—N5—C11	118.3 (4)	C10B—C9B—C12B	120.2 (17)
O6—N5—C11	118.1 (3)	C8B—C9B—C12B	118.2 (15)
C11—C6—C7	118.3 (4)	C9B—C10B—C11B	121 (2)
C11—C6—H6A	120.9	C9B—C10B—H10B	119.5
C7—C6—H6A	120.9	C11B—C10B—H10B	119.5
C6—C7—C8	123.1 (4)	C6B—C11B—C10B	121.0 (19)
C6—C7—N4	115.5 (4)	C6B—C11B—N5B	121 (2)
C8—C7—N4	121.3 (4)	C10B—C11B—N5B	118 (2)
O1—C8—C7	124.5 (4)	O3B—C12B—O2B	124 (2)
O1—C8—C9	120.6 (4)	O3B—C12B—C9B	119.1 (17)
C7—C8—C9	114.9 (3)	O2B—C12B—C9B	116.2 (17)
			
C5—N1—C1—C2	1.0 (5)	O6—N5—C11—C10	−173.2 (4)
N1—C1—C2—N2	174.7 (3)	C10—C9—C12—O3	−4.5 (5)
N1—C1—C2—C3	−1.9 (5)	C8—C9—C12—O3	175.3 (4)
N2—C2—C3—N3	4.7 (5)	C10—C9—C12—O2	176.3 (3)
C1—C2—C3—N3	−178.6 (3)	C8—C9—C12—O2	−3.8 (5)
N2—C2—C3—C4	−175.5 (3)	C11B—C6B—C7B—C8B	−10 (11)
C1—C2—C3—C4	1.2 (4)	C11B—C6B—C7B—N4B	178 (8)
N3—C3—C4—C5	−179.8 (3)	O4B—N4B—C7B—C6B	−170 (5)
C2—C3—C4—C5	0.4 (5)	O5B—N4B—C7B—C6B	1(7)
C1—N1—C5—C4	0.7 (5)	O4B—N4B—C7B—C8B	18 (6)
C3—C4—C5—N1	−1.4 (5)	O5B—N4B—C7B—C8B	−171 (4)
C11—C6—C7—C8	0.0 (17)	C6B—C7B—C8B—O1B	−176 (5)
C11—C6—C7—N4	−179.7 (9)	N4B—C7B—C8B—O1B	−5(6)
O4—N4—C7—C6	−164.1 (11)	C6B—C7B—C8B—C9B	10 (7)
O5—N4—C7—C6	16.1 (17)	N4B—C7B—C8B—C9B	−179 (4)
O4—N4—C7—C8	16.2 (18)	O1B—C8B—C9B—C10B	179 (4)
O5—N4—C7—C8	−163.6 (11)	C7B—C8B—C9B—C10B	−7(6)
C6—C7—C8—O1	178.0 (9)	O1B—C8B—C9B—C12B	8(5)
N4—C7—C8—O1	−2.3 (17)	C7B—C8B—C9B—C12B	−178 (3)
C6—C7—C8—C9	−3.0 (16)	C8B—C9B—C10B—C11B	4(10)
N4—C7—C8—C9	176.7 (10)	C12B—C9B—C10B—C11B	175 (8)
O1—C8—C9—C10	−177.5 (4)	C7B—C6B—C11B—C10B	7(16)
C7—C8—C9—C10	3.5 (9)	C7B—C6B—C11B—N5B	−172 (10)
O1—C8—C9—C12	2.7 (7)	C9B—C10B—C11B—C6B	−4(16)
C7—C8—C9—C12	−176.4 (7)	C9B—C10B—C11B—N5B	175 (9)
C8—C9—C10—C11	−1.0 (6)	O6B—N5B—C11B—C6B	9(19)
C12—C9—C10—C11	178.9 (4)	O7B—N5B—C11B—C6B	−167 (12)
C7—C6—C11—C10	2.8 (12)	O6B—N5B—C11B—C10B	−170 (11)
C7—C6—C11—N5	−178.3 (9)	O7B—N5B—C11B—C10B	15 (18)
C9—C10—C11—C6	−2.3 (7)	C10B—C9B—C12B—O3B	6(6)
C9—C10—C11—N5	178.7 (4)	C8B—C9B—C12B—O3B	177 (3)
O7—N5—C11—C6	−171.6 (5)	C10B—C9B—C12B—O2B	175 (4)
O6—N5—C11—C6	7.8 (7)	C8B—C9B—C12B—O2B	−14 (4)
O7—N5—C11—C10	7.3 (6)		

### Hydrogen-bond geometry (Å, °)

**Table:**

*D*—H···*A*	*D*—H	H···*A*	*D*···*A*	*D*—H···*A*
N1—H1N1···O1	1.07	1.76	2.753 (4)	153
O2—H2···O1	0.82	1.72	2.485 (4)	154
N2—H2N2···O6^i^	0.89	2.24	3.120 (4)	171
N2—H1N2···O3^ii^	1.03	2.11	3.026 (5)	146
N3—H1N3···O6^i^	0.89	2.36	3.104 (4)	142
N3—H2N3···O5^iii^	1.00	2.24	3.217 (5)	163
C6—H6A···O3^iv^	0.93	2.56	3.299 (6)	136

Symmetry codes: (i) *x*−1, *y*−1, *z*; (ii) −*x*+1, *y*−1/2, −*z*+1/2; (iii) −*x*, *y*−1/2, −*z*−1/2; (iv) *x*, −*y*+3/2, *z*−1/2.
